# Supplementary material for: Are carbon emissions trading and green financial instruments synergistic? -Comprehensive quantitative research based on content analysis
Source: PLoS One. 2024 Mar 7;19(3):e0298601. doi: 10.1371/journal.pone.0298601 (PMC10919601; doi:10.1371/journal.pone.0298601)
Supplement: S1 Data — (ZIP) [file pone.0298601.s001.zip › Data/地级市Z_F工作报告.zip!/╕ú╜¿/╞╬╠∩╩╨/└┤╫╘╣1⁄2╓┌║┼í╛┬φ┐╦╩2╛▌═°í┐_2007.docx]

各位代表：

现在，我代表莆田市第四届人民政府向大会做工作报告，请予审议，并请市政协委员和其他列席同志提出意见。

凝心聚力 奋力追赶 实现经济社会发展新跨越

六年来，在中共福建省委、省政府和中共莆田市委的坚强领导下，市政府团结依靠全市人民，高举邓小平理论和“三个代表”重要思想伟大旗帜，认真贯彻落实党的十五大和十六大精神，坚持以科学发展观统领全局，围绕建设海峡西岸经济区的战略部署，加快推进湄洲湾港口城市建设，圆满完成了“十五”计划和本届政府确定的各项任务。

国民经济快速增长，综合实力跃上新台阶。全市生产总值从2000年的183.86亿元增加到2006年的422.61亿元，总量从全省第九位上升到第七位，年均增长13.3%。财政总收入从13.51亿元增加到 37.3亿元，年均增长18.4%；其中地方级收入从8.57亿元增加到18.49 亿元，年均增长13.7%。规模以上工业产值从116.7亿元增加到482.9亿元，年均增长26.9%。全社会固定资产投资累计完成516亿元，是建市的1983年至2000年投资总和的1.5倍。金融运行良好，上年末人民币存贷款余额分别达到377.8亿元、228.6亿元，是2000年末的2.67倍和2.34倍。产业结构不断优化，三次产业比例从2000年的20.0∶45.3∶34.7调整为上年的13.2∶55.8∶31.0。农业产业化步伐加快，水产、水果、畜牧、蔬菜和食用菌五大主导产业不断壮大。整合提升五个经济开发区，促进产业集聚，制鞋、电子、食品、纺织服装等十大产业的产值占全市规模以上工业产值的95.4%。第三产业日益壮大，新型商贸业不断涌现，旅游、房地产成为服务业的重要增长点。实施名牌战略，拥有中国驰名商标5个、中国名牌产品6个、国家免检产品17个。

项目带动持续运作，发展后劲增添新动力。围绕项目带动持续攻坚，持续运作，持续提升，有效促进项目落地。目前已立项和在建的项目总投资超过1000亿元。着力实施民资回归工程，2003年6月至上年末，累计引进合同项目2231个，完成投资257.87亿元。建成木兰溪下游防洪一期工程、外渡引水隧道工程、澄峰围垦、南日岛风力发电场、秀屿3.5万吨级多用途码头等重大项目。推进LNG气电一体化、林浆纸一体化、金钟水利枢纽、仙游抽水蓄能电站、进口木材检疫除害处理区、国家级木材贸易加工示范区、莆田工艺美术城、木兰溪下游防洪二期工程、平海湾跨海供水应急工程和滨海大道等一批重大项目建设，为我市跨越发展注入强劲的动力。福厦铁路（莆田段）和莆秀高速公路顺利开工，向莆铁路将于今年上半年开工建设，湄洲湾港口铁路支线获得铁道部批准建设，拓展了湄洲湾港口的战略腹地。

城乡面貌显著变化，人民生活水平实现新提高。建成了荔园路、荔涵大道、延寿路等市政道路，建成区面积从2000年的25.75平方公里扩大到2006年的46.8平方公里,城市规模拓展到66平方公里。规划建设妈祖城，以及滨海、城港、荔港、仙港、涵港、沁峤路等通港大道，初步构建滨海港口城市框架。建设了城市污水、垃圾处理、管道燃气等城市公用设施，兴建了市体育中心、广电中心、莆田一中、二中新校区等社会公益设施，新建了东岩山、天九湾等公园，获得省级园林城市称号。建设了20多个新型社区，其中荔园小区为省城市住宅建设示范区。进一步完善农村水、电、路等基础设施，除险加固病险水利工程2750处，解决93.9万人群众的饮水困难，新增水电装机容量4.59万千瓦，铺设四级以上水泥路2500公里，行政村道路硬化率达到97.3%。开通农村客运线路249条，行政村通车覆盖率达到95%。开展农村“家园清洁行动”，村容村貌整治项目获得“中国人居环境范例奖”。城乡居民收入进一步提高，城镇居民人均可支配收入达到12274元，农民人均纯收入达到4922元，分别比2000年增长92.1%、51.3%。2006年末居民人民币储蓄余额达到272.45亿元，比2000年末增长1.5倍。

改革开放纵深推进，投资环境有了新改善。顺利调整行政区划，落实县区经济管理权限，县区经济更具活力。农村税费改革全面推行，集体林权制度改革顺利实施。国企改革基本完成，雪津啤酒国有股权成功转让，国有资产实现保值增值。众和股份有限公司在国内A股市场上市，实现我市企业国内主板上市融资零的突破。推进政府管理创新，实行重大事项由市政府常务会议研究决定制度，开通网上投诉和24小时市长热线电话，建立政府新闻发言人制度，推行行政执法责任制工作，取消、下放及改变审批方式635项，率先实施政府绩效评估试点工作。建立公共突发事件应急机制，有效应对非典、禽流感等重大疫情及特大风暴潮等自然灾害。外经贸工作不断加强，实际利用外资累计20.14亿美元，年均增长7.4%；外贸出口总额64.15亿美元，年均增长10.9%。推进与港澳台同胞、海外侨胞的经贸文化交流，创立中华妈祖文化交流协会，举办了每年一届的中国湄洲妈祖文化旅游节，2006年成功接待了7000多名台胞来莆谒祖进香团，实现湄洲岛与金门的海上直航。扩大国际交往，与美国贝茨威尔市和乌拉圭东角市签订了缔结友好城市意向。成立“一办三中心”，进一步优化投资环境，我市入围2006年跨国公司眼中“最具有投资价值的中国城市”候选名单，连续三年登上福布斯“中国大陆最佳商业城市榜”，连续三年成为台商投资值得推荐城市。

社会事业全面发展，和谐莆田谱写新篇章。实施科教兴市战略，首获“全国科技进步先进城市”称号，获得国家专利557项。巩固提高“两基”成果，适龄儿童入学率达到99.7%，初中入学率达到98%。涵江、城厢、荔城三个区和湄洲岛管委会通过“双高普九”验收。新增省一级达标校5所，成立了莆田学院和湄洲湾职业技术学院。一批文化精品在全国全省获奖，中央电视台“心连心”艺术团首次来莆慰问出并取得圆满成功，莆仙戏和妈祖祭典列入国家非物质文化遗产目录，湄洲妈祖祖庙等列入全国第六批重点文物保护单位。成功举办了第五届中国摄影艺术节、首届中国（莆田）海峡工艺品博览会，获得“中国摄影之乡”、“中国木雕之城”称号。实施村村通广播和电视工程，广播、电视覆盖率分别达到98.01%、98.09%。建立健全公共卫生体系，建成市疾控中心、市急救中心、市第五医院等一批医疗卫生设施。荔城、城厢、涵江率先实行新型农村合作医疗制度。体育事业蓬勃发展，获得全国“篮球城市”称号，成功申办第十四届省运会。营造良好的劳动就业环境，城镇新就业和下岗再就业人数分别达到3.4万人、1.6万人，转移农村劳动力76.4万人次。城乡居民最低生活保障制度全面实施，失业、医疗、养老、工伤和生育等保险稳步推进，社会保障体系逐步建立。认真落实人口与计划生育、国土资源、环境保护基本国策，促进人口与资源、环境和经济社会的协调发展。精神文明建设深入开展，获得省文明城市、省级卫生城市称号。“双拥”工作取得成效，连续两届被命名为“全国双拥模范城”。 “平安莆田”建设扎实推进，“四五”普法通过验收，信访工作得到加强，安全生产形势平稳，社会保持安定稳定，形成了风正气顺、人和业兴的局面。

2006年，我市经济社会发展实现“十一五”规划的良好开局。全市生产总值增长16%，增幅居全省第二位；农业总产值增长5.8%，增幅居全省第三位；规模以上工业产值增长26.7%，增幅居全省第四位；财政总收入增长26.6%，增幅居全省第三位，其中地方级财政收入增长26.9%，增幅居全省第四位；社会消费品零售总额增长13.8%，增幅居全省第五位；全社会固定资产投资额增长47.7%，增幅居全省第二位；外贸出口总额增长14.8%，增幅居全省第七位；实际利用外资（验资口径）增长42.4%，增幅居全省第二位；人口自然增长率6.8‰。

各位代表，过去六年的奋斗历程告诉我们：发展为大、发展为先、发展为重，任何时候都不能动摇；项目带动、民资回归、项目攻坚，任何时候都不能削弱；持续提升、持续运作、持续发展，任何时候都不能耽误；人民唯大、人民唯先、人民唯重，任何时候都不能忘记。

各位代表，过去六年，300万莆田人民奋力追赶，铁心拼搏，跨步跃进，创造了莆田发展史上最辉煌的业绩。成绩应该归功于党、归功于人民、归功于为我市建设付出心血和汗水的各界朋友。在这里，我代表市人民政府，向给予我们支持与监督的人大代表、政协委员，各民主党派、工商联和无党派人士、各人民团体、社会各界人士，向各位老领导、老同志，向驻莆部队指战员、武警官兵和公安干警，向所有关心支持莆田建设的港澳台同胞、海外侨胞和国际友人，向依法纳税人，致以崇高的敬意和衷心的感谢！

我们在肯定成绩的同时，也清醒地看到经济社会发展还面临一些困难和问题：港口优势还没有充分发挥，经济总量仍然偏小，经济实力依然不强；产业链不完善，产业集群不发达，高新技术产业比重低，第三产业发展明显偏弱，消费需求不足，经济结构不尽合理；城市建设管理水平比较低，功能还不够完善；区域发展不平衡，一些县区、乡镇财政依然困难；企业用工结构性矛盾比较突出，土地、能源、资金、人才等要素制约趋紧；不同群体的收入差距仍然较大，社会保障水平不高，农民持续增收难度较大，部分城乡群众生活还比较困难；安全生产隐患仍然存在，社会治安状况不尽人意；政府职能转变还不到位，依法行政、经济管理和办事效率有待进一步提高；个别部门和公务员责任心不强，服务态度差，“中梗阻”现象依然存在，抓落实、办实事、促发展的力度还不够大；极少数干部违法违纪，消极腐败现象仍然存在，廉政建设有待加强。对于这些问题，我们要高度重视，认真加以解决。

乘势而上 再创佳绩 开创湄洲湾港口城市新局面

今后五年是我市跨越发展的关键时期。新一届政府工作的指导思想是：以邓小平理论和“三个代表”重要思想为指导，认真贯彻党的十六届六中全会和即将召开的党的十七大精神，全面落实省第八次党代会和市第五次党代会的部署，牢固树立科学发展观，加快构建社会主义和谐社会，着力调整经济结构和转变增长方式，着力加强资源节约和环境保护，着力推进改革开放和自主创新，着力促进社会发展和解决民生问题，把重在持续、重在提升、重在运作、重在实效的实践要领贯穿于各项工作中，继续实施项目带动战略，继续实施民资回归工程，继续实施项目攻坚，发挥拓展一线的骨干作用，积极建设资源节约型、环境友好型、创新型城市，努力实现速度、质量、效益相协调，消费、投资、出口相协调，人口、资源、环境相协调，实现又好又快发展，加快推进湄洲湾港口城市建设进程。

今后五年，我们要加快建设产业集聚强劲、配套功能完善、生态环境优美的湄洲湾港口城市，实现“经济翻番、港城崛起”目标，即在优化结构和提高效益的基础上，地区生产总值年均增长15%以上，经济总量稳居全省第五位。建成经济发展强市、百万中心城区、水陆交通枢纽、滨海旅游胜地、制造加工基地、电力能源中心、特色种养之乡、工艺美术之城和新型人才高地，促进莆田在海峡西岸城市群中迅速壮大和崛起。

一是开发大港口。集全市之智，举全市之力，加快港口开发建设，把湄洲湾港建成东南沿海的大宗货物集散地、具有深水中转和战略物资储备等多功能的综合性枢纽港。抓好湄洲湾、兴化湾等港区开发，建设一批深水码头、专用码头和多用途码头，形成大中小港口相结合、大中小泊位相配套，规模化、集装箱化、信息化程度较高的港区开发体系。建成福厦铁路、向莆铁路和湄洲湾港口铁路支线，开通绕城高速和莆秀高速公路，筹建莆田机场和湄洲岛跨海通道，开辟一批国内外港口航线，形成承南接北、海陆空互补、快速便捷的交通运输体系。加快建设物流、仓储、信息、电力、供排水和燃气管道网等设施，形成适度超前、功能完善、设施齐全的综合配套体系。积极引进以炼化一体化项目为重点的石化产业，以船舶修造和冶炼项目为重点的冶金机械产业，以气电、火电和风电项目为重点的能源产业，以林浆纸一体化项目为重点的林产加工产业，以水泥项目为重点的建材产业，形成一批产业关联度强、港口吞吐量大、资金密集度高的临港产业体系。完善投融资、岸线开发、通关环境和港铁协作等港航开发体系。

二是打造大产业。加大对农业的投入，改善农业基础设施，推进农业科技创新，转变农业增长方式，大力扶持龙头企业，提高农业的企业化程度，加强莆台农业合作，加快发展现代农业。加快建设 “两个基地”，打造“四个产业集聚平台”，拓展“五个经济开发区”，推进LNG气电一体化、林浆纸一体化、抽水蓄能电站和核电站等一批重大项目。大力培育纺织服装、机械制造、医药医疗器械、工艺美术、能源、电子信息、食品加工、化工、制鞋和林产加工等“十个产业集群”，力争产值超过1000亿元，形成规模和品牌优势，建成海峡西岸重要制造业基地。着力打造现代服务业，做强做大第三产业。推进海峡西岸旅游胜地建设，深度开发旅游资源，大力拓展旅游市场，培育旅游经济。完善物流业发展规划，建设荔城黄石、涵江闽中、秀屿港口和仙游枫亭等物流园区，培育物流经济。加快会展平台建设，继续举办中国（莆田）海峡工艺品博览会、中国（莆田）木业投资贸易洽谈会、中国（秀屿）医疗器械药品展销会、中国（莆田）国际制鞋技术设备暨鞋材展览会，培育会展经济。提供优惠条件，集聚国内外大型企业集团采购中心、营运总部或分支机构，培育总部经济。抓住承办第十四届省运会契机，加快发展体育产业，带动第三产业发展，培育省运经济。重点推进海洋强市建设，加快兴化湾海洋经济开发贸易区、平海湾海洋经济综合开发区、南日岛生态经济区建设，发展海洋经济。

三是建设大港城。主动融入海峡西岸城市群，加快建设中心城区、滨海新城区、仙游城区，打造“一心九组团”的大城市格局。中心城区按照“提升中北部、拓展东西南”的思路，以片区改造为切入点，提升城市中部和北部的建设水平，向东、西、南三个方向拓展新区。推进妈祖城建设，力争三年完成基础设施建设，五年初具规模。融合湄洲岛国家旅游度假区，突出滨海旅游和妈祖文化特色，初步建成滨海新区，为构建海湾型城市奠定基础。加快建设鲤城、鲤南城区和枫郊组团，壮大仙游城区。着力推进“四纵八横”主干路网建设，建成火车站、汽车客运枢纽站，增加一批市区公交站和乡村客运站等交通设施，完善城市综合交通体系。推进城市供水、电力、燃气管道、垃圾污水处理和城市防灾体系等设施建设，建成“七馆二场”等体育场馆，以及市科技馆、莆仙大剧院、图书馆、博物馆等公益设施，完善城市功能。着力整治城区内河、护城河、南北洋河道，继续推进木兰溪下游防洪二期工程，加快木兰溪下游防洪三、四期工程和下游挡潮闸的前期工作，实现河畅、水清、岸绿。建立健全城市管理新机制，积极推行城市社区管理模式，提高城市管理水平。结合铁路、主干公路、开发区和城市建设拆迁安置，建成一批高标准的城市新社区，推进城乡一体化进程。

四是推进大开放。继续发挥湄洲湾港口和五个经济开发区招商引资的主渠道作用，争取设立莆田出口加工区、保税区等引资新平台，积极探索收购、兼并等引资新方式，鼓励外企特别是跨国公司、国际大财团投资高新技术产业、基础设施、农业和服务业，参与传统产业改造升级，提高利用外资水平。实施“科技兴贸、以质取胜”战略，培育壮大外贸出口企业，促进外贸出口市场多元化，提高外贸出口竞争力。鼓励企业到境外投资兴业，扩大劳务输出，提高经济外向度。继续举办世界莆商大会、世界兴安同乡恳亲会、妈祖文化旅游节和中国湄洲妈祖·海峡论坛。加快建设台商投资区、对台小额贸易集中区、莆台农渔业合作实验区、闽东南水产品物流中心、台湾农民创业园、闽台文化交流中心等，促进莆台合作。加强与海外侨胞联系，促进与港澳台的经贸文化交流合作，吸引莆商回乡投资创业，扩大非莆籍企业的投资规模。

五是促进大和谐。加强民主法制建设，坚持依法行政、民主行政、科学行政，强化社会管理和公共服务职能，推进决策科学化、民主化，深化政务公开，强化绩效评估工作，建设对人民负责的政府。坚持社会主义核心价值体系，加强新闻出版、广播影视、文学艺术、社会科学工作，培育温馨友爱、团结奋进的人文精神。开展创建和谐社区、和谐家庭等活动，形成人人促进和谐的局面。加快社会主义新农村建设，促进城乡协调发展。注重发展科技、教育、卫生、文化、体育等社会事业，促进经济社会协调发展。加强人口与计划生育工作，统筹解决人口问题，促进人的全面发展。加强生态环境保护，促进人与自然和谐。争创国家园林城市、中国优秀旅游城市、国家环保模范城市。争取蝉联“全国双拥模范城”和“全国科技进步先进城市”称号，确保成功承办第十四届省运会。实施积极就业政策，构建和谐的劳动关系。推进养老、医疗、失业、工伤、生育等保险制度建设，发展社会保险、社会救助、社会福利、慈善事业，探索解决农村养老、失地、失海农民保障问题。发展残疾人、老龄事业。实施“五五”普法规划，落实民族、宗教和侨务政策，加强社会治安综合治理，建设“平安莆田”。重视信访工作，强化安全生产责任制，完善各类应急预案。深化军民共建工作，增强国家安全意识，加强国防建设。

重在持续 重在提升 推动2007年经济社会新发展

今年我市经济社会发展的主要目标是：全市生产总值突破500亿元，增长16%；工业增加值253亿元，增长20%；规模以上工业产值605亿元，增长24%；财政总收入42亿元，增长12.6%，其中地方级财政收入21亿元，增长13.6%；社会消费品零售总额167.8亿元，增长15%；全社会固定资产投资额225亿元，增长36.2%；外贸出口总额14.5亿美元，增长13.3%；实际利用外资5.5亿美元，增长19.8%；城镇居民人均可支配收入增长8%；农民人均纯收入增长7%；城镇登记失业率控制在3%以内；人口自然增长率控制在7.5‰以内；单位生产总值能耗降低4%；主要污染物排放总量减少1.5%。

为了实现以上目标，我们要重点抓好以下工作：

（一）加快港口开发，促进持续发展

推进港航建设。完成《莆田市港口总体规划》和港口岸线控制性规划修编。加强招商引资，吸引国内外知名航运企业投资建设港航设施，开通新的国际航线。续建福建LNG10万吨级码头、秀屿4万吨级木材专用码头、湄洲湾公用航道一期工程，新建秀屿两个5万吨级多用途码头、秀屿5万吨级和万吨级石油化工码头，推进东吴1#-3#泊位、林浆纸一体化两个15万吨级码头及两个3万吨级配套码头建设和罗屿25万吨级散货码头前期工作。疏浚湄洲湾25万吨级、莆头段5万吨级和东吴15万吨级三条航道，改善通航条件。

构建疏港通道。全面完成福厦铁路征地拆迁任务，加快工程建设进度。动工建设向莆铁路、湄洲湾港口铁路支线。加快建设莆秀高速公路，贯通城港、荔港、仙港、涵港、沁峤路和省道201线，新建省道202线。做好绕城高速公路和湄洲岛跨海通道前期工作。

发展临港工业。依托湄洲湾石化基地的东吴和枫亭石化工业区、三江口化工园区、秀屿国家级木材贸易加工示范区及飞地工业园区，大力引进临港项目。加快福建LNG接收站、莆田燃气电厂、中原港务30万立方米中心油库等一批临港重点项目建设，扩建南日岛风电工程，动工建设LNG空气分离冷能利用项目，推进LNG二期、火电厂二期、海螺水泥中转加工、东源及联发船舶修造等项目前期工作。

（二）加大项目攻坚力度，增强发展后劲

抓好重大项目建设。千方百计抓好105个在建重点项目、55个预备重点项目和57个重点前期项目，确保完成年度投资任务，提高重点项目投资占固定资产投资的比重。续建仙游金钟水利枢纽、木兰溪二期防洪工程、莆田管道燃气、莆田220KV主干电网、莆田学院扩建、妈祖城和平海湾跨海供水应急工程等重点项目，动工建设仙游抽水蓄能电站、罗屿公铁两用大桥和东圳灌区配套工程，深化莆田机场、南北洋排涝等重点工程前期工作。

完善项目工作机制。围绕我市“十一五”规划和十大产业集群，策划生成一批基础设施、重点产业、社会事业等事关莆田长远发展的重大项目。加大项目推介力度，扩大引进外资规模，吸引民资回归，鼓励现有企业做强做大。注重动作，抓好企业技改、资产重组，特别是上规模、有效益、高质量的项目，争取更多的项目列入国家、省重点建设盘子。进一步落实重点项目领导挂钩工作责任制，加强项目协调服务，完善工程建设项目招标投标制度，优化项目建设环境。

突破发展要素制约。修编土地利用总体规划，科学合理安排各项用地，确保重点项目、工业项目和社会事业项目用地。盘活闲置土地和厂房，鼓励发展标准厂房，建造多层厂房，拓展项目用地空间。破解企业用工难题，开展省际劳务协作，完善劳动力供求信息网络，规范劳动力市场，抓好企业用工培训，实行外来员工市民待遇，加强劳动监察，维护外来劳动者的合法权益。强化资金、技术、人才保障，做好煤、电、油和火工材料等物资调度，确保项目顺利推进。

（三）加快建设制造业基地，提升发展质量

培育壮大产业集群。实行领导干部挂钩产业集群责任制，培育壮大十大产业集群。加强经济开发区的水、电、路、通讯、排污等基础设施和生活服务区建设，引导产业集聚。加快雪津啤酒80万吨技改、天喔食品等一批项目建设进度，推动才子、金威、云敦等服装企业扩大生产规模，促进合众天成、众和纺织面料等项目投产达产，重组提升制鞋、食品、纺织服装等传统优势产业。推进莆田工艺美术城建设，举办第二届中国（莆田）海峡工艺品博览会，扶持工艺美术产业。加快木材加工区建设，促进已签约项目动工建设，跟踪落实莆籍在沪、在京企业投资团入驻国家级木材贸易加工示范区，推进原料林基地建设，开工建设林浆纸一体化项目，深化配套林产加工产业。加快秀屿医药医疗器械科技城建设，力促一批项目落地，发展医药医疗器械产业。推进PT液晶显示器、光纤陶瓷等项目落地，加快安特6英寸集成电路芯片项目建设，支持新威电子、台湾通信、德信电子等企业增资扩产，壮大电子信息产业。

做强做大骨干企业。进一步提高工业投资占固定资产投资和工业项目占重点建设项目的比重，重点抓好46个重点工业项目和50个新增产值3000万元以上企业的建设。重点扶持112家产值超亿元的骨干企业，培育税收贡献大、市场竞争力强的企业集团。积极支持有条件的企业上市融资，扩大规模，增强竞争力，同时，扶持发展中小企业。继续实施品牌战略，再创一批中国驰名商标、中国名牌产品、国家免检产品和省著名商标、省名牌产品。

增强企业创新能力。推进一批工业结构调整重点项目，实施一批自主创新项目，支持一批企业通过ISO国际质量管理体系认证。落实《莆田市奖励企业技术（研发）中心建设规定》，鼓励企业创建设计研发中心、质检中心、创新服务中心，力争获得一批国家级和省级企业技术中心。鼓励企业自主创新、合作创新和引进消化吸收再创新，扶持发展民办科研机构，鼓励外资、合资企业在莆设立研发机构，建设制鞋等行业技术开发基地，促进“莆田制造”逐步走向“莆田创造”。

（四）提升服务业水平，培育发展新增长点

发展旅游产业。围绕打造滨海旅游胜地目标，开发海岛、生态、文化、红色旅游资源，带动沿海、平原、山区旅游协调发展。重点抓好湄洲岛国家旅游度假区建设，充分发挥九鲤湖、南少林、广化寺等旅游资源的作用，开发建设一批新景点，推进高级酒店建设，完善旅游接待设施。深化莆台旅游合作，继续推动湄洲－金门旅游直航，建设台货小商品市场，举办海峡旅游商品展销会，构建环海峡妈祖信仰旅游圈。

发展房地产业。控制经营性土地市场投放规模，全面清理未开发经营性土地，认真解决土地增值税政策实施中可能产生的问题，强化房地产监管，促进房地产市场可持续发展。严格按照规划，建成一批新型商住小区，完善廉租房制度，规范经济适用房建设，解决中低收入人群住房困难。

发展服务业。结合旧城改造和新区建设，建成一批商贸物流中心、高档写字楼，引进沃尔玛、乐购等世界知名企业入驻莆田。加强特色商业街规划建设，逐步培育餐饮娱乐、品牌服装、装潢材料、汽车配件、旅游购物等一条街。推广连锁经营、代理制、配送制等现代营销方式，培育一批大型连锁龙头企业。积极培育文化娱乐、体育休闲、医疗保健和电子信息等服务业，大力发展金融、会计、律师、公证、保险、咨询、电信等服务业，加快发展设计研发、产品检测等生产性服务业，促进服务业与工业互动发展。

扩大消费需求。实施“万村千乡”市场工程，新建一批合格农家店，健全农村消费网络，促进农村消费。加快城乡专业市场建设，开工建设莆田鞋业服装城。发展社区服务业，扩大社区居民购物、餐饮、保健、休闲等领域消费。加强市场监管和消费者维权工作，营造良好的市场环境。

（五）深化改革开放，增强发展活力

扎实提升对外开放水平。积极参加“5·18”、“6·18”、“9·8”等各种招商活动，举办莆商大会、妈祖文化旅游节，争取设立莆田出口加工区，多形式开展招商引资，吸引世界500强企业、跨国公司和中直企业来莆投资，鼓励外资并购我市企业股权或购买外企中方股权，提高外资利用质量。健全招商引资工作责任制和激励机制，强化项目跟踪服务，实行投资千万美元以上的外资项目专人联系制度，提高项目履约率。开拓国际市场，加快外贸出口调整，努力实现外贸进出口的两个根本转变，增强外贸出口竞争力。扩大民营出口经营主体，新增一批从事对外贸易的民营企业。实施“走出去”战略，鼓励有条件的企业到国外投资办厂、承包工程和劳务输出。优化通关环境，提高通关效率。

继续实施民资回归工程。策划生成一批民资回归投资项目，赴东南亚、港澳台地区和北京等莆商聚集的城市进行项目推介，吸引莆籍企业家联合组团回乡投资创业。积极鼓励民资投资基础设施、重点产业和社会事业，更好地发挥“民资回归”的带动作用。建立健全民营企业创业辅导、信用担保、人才培训等服务体系，及时解决民资回归中的困难和问题，扶持一批民营企业进入规模以上企业行列。组织民企参加各种贸洽会、展销会等经贸活动，开拓莆货市场，提升莆货知名度。

大力深化各项改革。深化农村税费配套改革，推进乡镇机构改革试点工作，完善农村义务教育管理体制，改革县乡财政管理体制，坚持农村基本经营制度，加快征地制度改革。深化农村集体林权制度改革，明晰产权，放活经营，发展林业。完善国有资产监督管理体制，建立健全国有资本经营业绩考核和激励约束机制，实现国有资产保值增值。深化文化体制改革，促进文化事业和文化产业发展。深化行政审批制度改革，减少和规范行政审批事项，降低行政成本。推进事业单位改革。开展小城镇机构改革试点工作，赋予经济发达的镇一级政府更多的经济管理和社会管理权限。深化投资体制改革，继续推行业主法人招标和代建制、政府投资公示和重大投资项目后评估制度。

（六）做好财税金融工作，壮大地方财源

加强财税征管。抓好重点企业、重点税源监控，严厉打击偷逃骗税行为。加强罚没收入、政府资源性收入等非税收入的征管，规范行政事业性收费管理。推进公共财政体制改革，整合财政各类收入，强化资金运作能力，集中财力办大事，确保重点支出。完善预算编制改革，深化部门预算和政府收支分类改革，扩大国库集中支付试点工作。全面清查行政事业单位资产，统筹监管非经营性国有资产。加强各类基金的收支监管。防范化解政府债务风险，逐步解决镇村财务困难。

培育地方财源。发挥财政资金引导和带动作用，支持产业发展，壮大骨干财源。大力发展餐饮服务、建筑安装、交通运输、旅游等第三产业，规范土地出让金收支管理，选择一批市政设施和各类特许经营权进行招投标和公开拍卖，增加地方财政收入。开展纳税大户评比表彰活动，培植一批税收大户，增强财政发展后劲。

提高金融服务水平。支持金融创新，加强银企合作，引导金融机构支持产业集群、中小企业发展和基础设施重点项目建设。推进林权、海域使用权抵押贷款，增加金融支农力度。加大下岗失业人员小额担保贷款、劳动密集型小企业贴息贷款。扩大汽车、住房消费信贷。

（七）促进资源节约和环境友好，转变发展方式

建设创新型城市。着力实施建设创新型城市的决定，认真做好“全国科技进步先进城市”二连冠迎评工作。继续实施“三大科技发展工程”，加快建设“六个科技示范基地”。开展院地科技合作，引进一批重大高新技术项目，开发一批具有自主知识产权的高科技产品，壮大高新技术产业。继续实施人才强市战略，充分发挥莆籍院士、专家、学者的重要作用，培养和引进一批适用人才、专门人才和拔尖创新人才。加大打假和反盗版力度，保护知识产权。加快“数字莆田”建设，提高信息化水平。

发展循环经济。严格执行循环经济发展总体规划，滚动实施“1520”节能示范工程，控制高能耗项目，禁止高污染项目，淘汰落后的生产工艺和设备，扶持发展无污染或少污染、能耗低、效益高的资源节约型产业。改造传统工业，重点抓好制鞋、造纸、电子、食品、机械制造等行业技改和治污工作。抓好重点领域和重点项目的环境保护工作，推行清洁生产，形成一批示范企业、园区。减少资源消耗和污染物排放，回收利用各类废弃物，实现资源循环利用。加快气电、风电建设，优化能源消费结构。开展全国节水型社会建设试点工作。

推进生态市建设。落实市长环保目标责任书年度任务。继续实施生态市建设总体纲要，积极创建一批生态示范区、示范镇、示范村，重点建设萩芦溪沿岸生态农业示范区、钟山生态农业观光园、秀屿区节水农业项目和湄洲生态岛等。严格基本农田保护，严厉打击违法用地行为。合理开发利用水资源，建立健全流域补偿机制，着力加强木兰溪上游、萩芦溪和东圳库区等生态环境保护。加强海洋生态环境保护，合理开发海洋资源。综合整治矿山环境，大力治理“青山挂白”现象。继续推进城乡绿化一体化和绿色通道工程建设，加强森林资源培育与保护，加快商品用材林、珍贵树种用材林等基地建设。

（八）统筹城乡建设，促进协调发展

推进中心城市建设。树立经营城市理念，盘活城市资产。完善城市中心区和近期发展地段控制性规划。加快中心城区道路网络建设，延伸文献东路、北大路、涵江人民街，打通镇海路、南园路、涵江河滨路和南环城路，加快建设下店路、东园路。加快推进妈祖城基础设施建设。继续实施荔园路三期工程，改造学园中路、福厦路市区路段。实施城市“新洁亮绿”工程，加快建设停车场、网络通讯、防灾减灾等城市配套设施，推进垃圾进场、污水进管。建设秀屿污水处理、闽中污水处理厂二期、黄石污水处理厂、市生物质能源发电厂、仙游污水和垃圾无害化处理等项目。

推进新农村建设。按照“生产发展、生活宽裕、乡风文明、村容整洁、管理民主”的要求，围绕市委提出的“十个目标”，大力拓展城市支持农村、工业反哺农业的渠道，积极抓好100个新农村建设试点。认真做好第二次全国农业普查工作。巩固农业基础地位，发展现代农业，培育60个农业产业化龙头企业，培育一批农产品品牌。落实粮食生产扶持政策，稳定粮食播种面积。继续实施 “六千”水利工程，改善农业生产条件。进一步完善水、电、路、陆岛码头、广播电视、通讯等农村基础设施建设，基本实现行政村道路硬化目标。实施农村“家园清洁行动”，重点整治500个行政村的村容村貌，改善农村人居环境。推进石城国家一级渔港、平海国家级僧帽牡蛎原种场、市级环境监测站综合实验室、渔业安全救助指挥系统建设，提高渔业生产基础设施配套水平。大力发展鲍鱼、龙须菜、海参等优势水产品养殖，促进鱼、贝、藻水产加工项目投产，做大海洋养殖业和海产品加工业。新转移农村富余劳动力4万人，增加农民收入。

推进区域协调发展。加大对经济欠发达县区的扶持力度，建立市对县区、乡镇财政发展的激励机制，增强县区、乡镇自主发展能力。仙游要强化沿海意识，融入湄洲湾，对接闽东南，发展工业经济、现代农业和旅游经济。荔城、城厢要加快建设中心城区，增强集聚辐射带动能力。涵江要培育壮大高新技术产业，发展传统商贸和旅游等第三产业。秀屿要依托港口和民资优势，发展临港工业、海洋经济和特色产业。湄洲岛要发挥妈祖文化和滨海旅游资源优势，为促进祖国和平统一服务。

（九）构建和谐社会，共享发展成果

健全民主法制。加快建设法治政府，自觉接受人大及其常委会的法律监督和工作监督，自觉接受政协的民主监督，认真办理人大代表议案、建议和政协提案。广泛听取各民主党派、工商联和无党派人士的意见，充分发挥工、青、妇等人民团体的桥梁纽带作用。加强城市社区建设，完善企事业、村（居）事务、财务公开和民主管理制度。积极开展法律援助，加强法律服务。

加强社会保障。落实就业再就业优惠政策，新增城镇就业2万人，新增下岗失业人员和就业困难对象再就业2000人。扩大各类社会保险覆盖范围，严格社会保障基金的征缴和管理。着力解决拖欠农民工工资问题，保障农民工的合法权益。提高农村居民最低生活保障标准，全面实行新型农村合作医疗制度，对农村困难家庭实行救助。加快安置房建设步伐，改善拆迁户居住条件。足额发放土地补偿金，保障失地、失海农民的利益。

发展社会事业。深入开展群众性精神文明创建活动，认真做好全省创建文明城市复评工作。更加注重教育的普惠性，大力扶持农村薄弱校建设，加快农村中小学危房改造和寄宿制学校建设，落实农村义务教育经费保障机制，推进义务教育均衡发展。做好城镇中小学布局规划，扩大优质高中教育资源。抓紧规划建设莆田一中妈祖城校区、莆田东方职业技术学院。大力发展中等职业教育，开展创建国家级、省级示范职校活动，多形式进行职业技能培训。加大家庭贫困学生的资助力度。重视幼儿教育和特殊教育。认真做好莆田学院、湄洲湾职业技术学院迎接教育部评估的各项工作。健全突发公共卫生事件应急体系，提高重大疾病预防控制、公共卫生监督和医疗救助能力。开工建设莆田学院附属医院新院区、市皮肤病防治医院新院区、建成市第一医院惠妹外科大楼、莆田人民医院，改造提升9所乡镇卫生院。大力发展城市社区卫生服务，健全农村三级服务网络。加强人口与计划生育工作，稳定低生育水平，提高人口素质。深入开展全民健身活动，提高竞技体育水平，积极筹备十四届省运会，加快市体育中心和城乡体育设施建设。扎实推进农村电影“2131”工程和广播电视“村村通工程”。加快建设莆仙大剧院，认真做好市博物馆、科技馆、图书馆、福利院等公益设施项目前期工作，继续加大妈祖文化宣传力度，建成妈祖阁和妈祖文化研究院，筹建妈祖乐府，组建福建省莆仙戏剧院，抓好莆仙戏和妈祖文化申报“世遗”前期工作。举办中国（莆田）南北少林武术公演盛会。继续做好审计、物价、人防、水文、气象、防震减灾、对台、扶贫、民族宗教、外事侨务、地方志、档案、妇女儿童和科普等工作。

维护社会安定。严格落实社会治安综合治理责任制，进一步推进“平安莆田”建设。加强公安“三基”工程建设，依法严厉打击各种刑事犯罪，特别是“两抢一盗”等侵财性和暴力性犯罪及“六合彩”赌博活动，加强社会治安防控体系建设，维护社会安定稳定。落实安全生产责任制，维护人民群众生命财产安全。加强信访和社会矛盾纠纷排查调处，发挥人民调解员的作用，及时化解人民内部矛盾。建立健全各类突发性事件的预警和应急处置体系，提高危机管理和抗风险能力。进一步提高国防意识，深入开展“双拥共建”活动，加强军政、军民团结。

创新管理 务求实效 加快建设服务型政府

服务是政府的天职。建设对人民负责的政府必须强化全心全意为人民服务的理念，落实“四个重在”实践要领，加强“四种能力”建设，更加注重大局，更加注重提速，更加注重破解，更加注重落实，努力使政府成为优质公共产品的提供者、良好经济社会环境的创造者、广大人民利益的维护者。

增强服务理念。政府的一切权力都是人民赋予的，一切属于人民，一切为了人民，一切服务人民。各级政府要按照建设为民、务实、清廉政府的要求，规范行政权力运行，牢固树立为经济发展服务，为社会公众服务，为企业和纳税人服务的执政理念，为人民掌好权，用好权。政府工作部门要不断提高服务能力、服务质量，以政府的优质服务促进全社会的优质服务，提高政府的执行力和公信力。全体公务员要竭诚为人民服务，在服务中增长才干，在服务中发挥智慧，在服务中实现人生的价值。

转变服务职能。切实提高统筹经济社会发展的能力，着力抓好经济调节、市场监管、社会管理和公共服务，切实把政府经济管理职能转到主要为市场主体服务和创造良好发展环境上来。着力解决就业、就学、就医、环境保护、社会治安等人民群众关心的社会问题；着力解决土地征用、拆迁安置、企业改制和下岗再就业等人民群众反映强烈的问题，坚决纠正损害群众利益的不正之风，为人民群众诚心诚意办好事，尽心竭力解难事，坚持不懈做实事。

创新服务方式。建立健全重大事项集体决策、专家咨询、社会公示与听证、评估论证等制度，推进政府决策科学化、民主化。积极推行办事公开，严格落实五项公开制度。完善政府新闻发言人制度，及时发布重大政务信息。加强“一办三中心”建设，简化审批环节，提高办事效率。加快电子政务和政府网站建设，建立综合公共服务平台，提供方便、快捷、高效的网上服务，降低行政成本。积极推进和规范公共资源市场化配置工作。严格执行工程项目招投标、土地使用权出让、产权交易和政府采购等公开制度。推行行政综合执法，实行相对集中行政处罚权改革。

提高服务效能。大力弘扬胡锦涛总书记倡导的八个方面的良好风气，强化公仆意识，全面加强政风建设，深入开展政府绩效评估工作和机关效能建设，从严治政。要严格执行市委做出的决策，不打折扣，不敷衍塞责，确保高度统一。要强化主动服务，对项目审批、项目建设、企业改制、招商引资等工作要主动介入，超前服务，凡是需要审批、协调、沟通的要提前告知，能够现场办理的马上就办。加大反腐倡廉力度，落实党风廉政建设责任制，继续开展治理商业贿赂，严厉打击以权谋私、钱权交易等行为。各级政府领导干部和每一位公务员都要忠于职守，奉公守法、秉公用权，防止行政不作为、乱作为，防止个别掌握有审批权、执法权的部门或个人通过既定的办事程序谋取私利。

各位代表！回首过去，我们豪情满怀；展望未来，我们信心百倍。我们要倍加珍惜我市当前来之不易的大好形势，倍加珍惜风正气顺、人和业兴的良好局面。当前，莆田人民强市富民的愿望无比强烈，攻坚克难的意志无比坚强，谋事创业的智慧无比丰富。我们一定要在中共莆田市委的坚强领导下，统一思想，坚定信心，真抓实干，勇往直前，奋力开创湄洲湾港口城市建设新局面，共同创造莆田更加美好的未来!
